# Flight speed and performance of the wandering albatross with respect to wind

**DOI:** 10.1186/s40462-018-0121-9

**Published:** 2018-03-07

**Authors:** Philip L. Richardson, Ewan D. Wakefield, Richard A. Phillips

**Affiliations:** 1 Department of Physical Oceanography, MS#21, Woods Hole Oceanographic Institution, 360 Woods Hole Road, Woods Hole, MA 02543 USA; 2 Animal Health and Comparative Medicine, University of Glasgow, Institute of Biodiversity, Graham Kerr Building, Glasgow, G12 8QQ UK; 3 British Antarctic Survey, Natural Environment Research Council, High Cross, Madingley Road, Cambridge, CB3 0ET UK

**Keywords:** Soaring, Dynamic soaring, Seabird, Airspeed, Ground speed, GPS-tracking, Bird flight performance, Flight polar diagram, ECMWF, Model

## Abstract

**Background:**

Albatrosses and other large seabirds use dynamic soaring to gain sufficient energy from the wind to travel large distances rapidly and with little apparent effort. The recent development of miniature bird-borne tracking devices now makes it possible to explore the physical and biological implications of this means of locomotion in detail. Here we use GPS tracking and concurrent reanalyzed wind speed data to model the flight performance of wandering albatrosses *Diomedea exulans* soaring over the Southern Ocean. We investigate the extent to which flight speed and performance of albatrosses is facilitated or constrained by wind conditions encountered during foraging trips.

**Results:**

We derived simple equations to model observed albatross ground speed as a function of wind speed and relative wind direction. Ground speeds of the tracked birds in the along-wind direction varied primarily by wind-induced leeway, which averaged 0.51 (± 0.02) times the wind speed at a reference height of 5 m. By subtracting leeway velocity from ground velocity, we were able to estimate airspeed (the magnitude of the bird’s velocity through the air). As wind speeds increased from 3 to 18 m/s, the airspeed of wandering albatrosses flying in an across-wind direction increased by 0.42 (± 0.04) times the wind speed (i.e. ~ 6 m/s). At low wind speeds, tracked birds increased their airspeed in upwind flight relative to that in downwind flight. At higher wind speeds they apparently limited their airspeeds to a maximum of around 20 m/s, probably to keep the forces on their wings in dynamic soaring well within tolerable limits. Upwind airspeeds were nearly constant and downwind leeway increased with wind speed. Birds therefore achieved their fastest upwind ground speeds (~ 9 m/s) at low wind speeds (~ 3 m/s).

**Conclusions:**

This study provides insights into which flight strategies are optimal for dynamic soaring. Our results are consistent with the prediction that the optimal range speed of albatrosses is higher in headwind than tailwind flight but only in wind speeds of up to ~ 7 m/s. Our models predict that wandering albatrosses have oval-shaped airspeed polars, with the fastest airspeeds ~ 20 m/s centered in the across-wind direction. This suggests that in upwind flight in high winds, albatrosses can increase their ground speed by tacking like sailboats.

## Background

Despite flapping their wings infrequently, wandering albatrosses (*Diomedea exulans*) routinely fly extremely long distances, even around the Southern Ocean [1]. They are believed to accomplish these feats primarily through the use of dynamic soaring, a phenomenon that has intrigued physicists and biologists for well over a century [2, 3]. Although numerous wandering albatrosses have been tracked in recent years [4–6], the extent to which their flight performance (i.e. airspeed and ground speed) varies under different conditions is not well known. This is because albatross flight maneuvers are complex and are employed primarily in remote areas of the Southern Ocean, making them difficult to observe and measure directly. Moreover, albatross ground speed varies with wind speed, flight directions relative to the wind direction (hereafter relative wind direction), and with different combinations of wind waves and swell waves [7–10]. By analyzing individual tracks, it has been demonstrated that the ground speed of albatrosses is dependent on the wind speed and wind direction relative to the flight direction. However, it remains unclear how variation in airspeed (the speed of a bird through the air) and in leeway (the downwind advection of a bird by the wind) interact to cause this relationship. Moreover, it is not well known how wandering albatrosses vary their airspeeds and ground speeds in wind speeds above the ~ 3–4 m/s minimum required for sustained dynamic soaring [11–13].

**Fig. 1:**
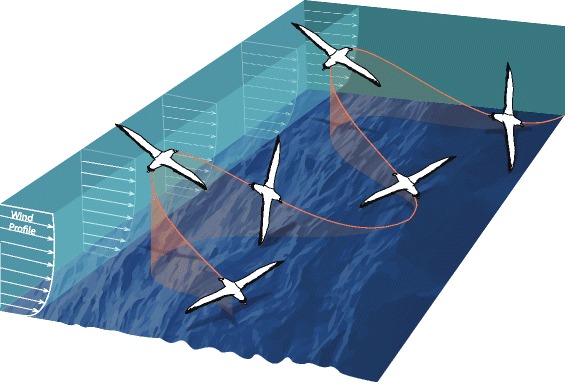
Schematic diagram showing an albatross flying in an across-wind direction using an S-shaped dynamic soaring maneuver (redrawn from [13]). The bird is shown soaring through a vertical profile of mean wind velocity. The bird extracts energy from the wind by climbing headed upwind and descending headed downwind. Significant waves are typically observed in the Southern Ocean. Wind-wave interactions cause a more complicated instantaneous wind field than that plotted here. Albatrosses appear to efficiently exploit the instantaneous in-situ winds and waves in dynamic soaring

Dynamic soaring exploits the vertical gradient of wind velocity over the ocean to obtain sufficient energy to sustain soaring flight [7, 12–14]. The typical flight pattern for across-wind dynamic soaring is an S-shaped maneuver, consisting of alternating upwind and downwind 90° turns (Fig. 1) [13]. For example, a bird climbs from close to the ocean surface in a wave trough diagonally upwind across the wind-shear layer to a height of around 10 m, turns ~ 90° downwind, descends diagonally across the wind-shear layer into a wave trough, and then turns ~ 90° into the wind again. Using this technique, wandering albatrosses are able to soar at speeds up to ~ 20 m/s in an across-wind direction. They probably also exploit updrafts over waves generated by the upward movement of the ocean surface and by wind-wave interactions to gain energy for soaring, especially at low wind speeds [7].

There is growing interest in the flight characteristics of albatrosses and other seabirds that use dynamic soaring (all medium to large Procellariiformes, as well as many sulids, large gulls, etc.), because this mode of flight provides a direct link between climate and biological processes [15–18]. For example, shearwaters undertaking trans-equatorial migrations are constrained to follow least-cost pathways defined by the global wind patterns [19–21]. In addition, recent increases in the foraging range and breeding success of wandering albatrosses are thought to have been mediated by strengthening winds in the Southern Ocean [22]. Similarly, variation in annual survival, breeding probability or breeding success of wandering albatross, black-browed *Thalassarche melanophris* and grey-headed albatross *T. chrysostoma* at South Georgia have been linked to changes in the wind regime [23], suggesting that ongoing global climate change may have major impacts on albatrosses, and pelagic seabirds in general. A necessary precursor to understanding and predicting how the wind affects the movements and, ultimately, the demography of seabirds is to quantify its effects on flight performance (i.e. flight speed).

Theory suggests that flying birds regulate their airspeed in relation to wind speed and relative wind direction. Firstly, it is hypothesized that birds proceeding at their maximum range speed (the speed at which most ground is covered per unit energy expenditure) should increase their airspeed in upwind flight [24, 25], although it has been argued that this hypothesis does not apply to soaring birds because their rate of energy expenditure is independent of airspeed [10]. Secondly, optimal foraging theory also predicts that birds searching for prey should increase their airspeed in headwind flight [10]. Empirical studies of variation in albatross airspeed with respect to flight direction relative to the wind are contradictory. Three species (wandering albatross, black-browed albatross and grey-headed albatross) tracked optically from land increased airspeed in headwind flight [26], while black-browed, grey-headed and Atlantic yellow-nosed albatross *T. chlororhynchos* tracked optically and by radar from a ship did not [10].

Polar diagrams of horizontal flight speeds are typically used to investigate avian flight performance with respect to wind speed and flight direction relative to the wind [10]. Accurate flight polars are necessary to parameterize mechanistic models of seabird flight, which in turn are needed to understand how migratory corridors and seabird demography are affected by climate change. The central aim of this paper is therefore to develop flight polars that quantify the effects of wind on albatross flight speed.

The best available summary of albatross ground speeds is a polar diagram [10], which includes 57 velocities of black-browed, grey-headed, and Atlantic yellow-nosed albatrosses measured by optical range finder [9]. Although this diagram includes ground speeds observed in various wind speeds and relative wind directions, the numbers of observations are fairly low and airspeed was not analyzed. More recently, tracking data from albatrosses fitted with satellite-transmitters (Platform Terminal Transmitters; PTTs) and GPS loggers were analyzed [8]. However, these data were not used to estimate the variation in airspeed with wind speed or to generate airspeed and ground speed polar diagrams. Although some high resolution GPS tracks of wandering albatrosses have been obtained [4, 27], only four measures of ground velocity and the associated wind velocity values have been published [27]. It therefore remains unclear to what extent the airspeed and ground speed of albatrosses varies as a function of the wind velocity.

Here, we develop empirical additive linear models to describe how flight performance of the wandering albatross varies under differing wind and flight conditions. Previous studies suggest that both ground speed and airspeed may vary with sex, wind speed and relative wind direction [8, 9, 28]. We extend these studies by analyzing GPS data at higher spatial and temporal resolution, and of higher accuracy (accurate to ~ 10 m, at 30–120 min intervals) collected from breeding wandering albatrosses. We estimated leeway velocity and calculated air velocity by subtracting leeway velocity from GPS-measured ground velocity. We then model the variations in airspeed and ground speed as functions of the speed and relative direction of the wind. Results are presented as modeled airspeed and modeled ground speed polar diagrams for six different values of wind speed from 3 to 18 m/s. Using these models, we test the hypotheses that: (1) the airspeed of albatrosses increases with wind speed, and (2) albatrosses increase airspeed in upwind flight relative to that in downwind flight.

## Methods

### Data collection

The albatross tracking data, and the extraction and manipulation of data on wind speed and direction are described in detail in [8]. In brief, 24 male and 22 female wandering albatrosses breeding on Bird Island, South Georgia (54.0°S, 38.0°W) were tracked by GPS during foraging trips made between February to September 2004. Birds were caught at the nest and BGDL-II GPS loggers (mass 67 g, dimensions 42 × 71 × 31 mm) [29] were attached to their mantle feathers using Tesa® tape. In addition, an activity logger recording saltwater immersion (British Antarctic Survey Mk IIa–IV loggers, either 5 or 10 g) [30] was attached to a plastic ring placed around the tarsus. Total instrument mass, including attachment materials, was 0.6% of mean body mass, well below the 3% limit recommended for biologging studies on seabirds [31]. GPS loggers recorded locations to an accuracy of ≤10 m, at a temporal resolution of 2 h during incubation, 30 min during brood-guard and 60 min during post-brood chick-rearing. Activity loggers tested for saltwater immersion every 3 s and recorded a value between 0 and 200, representing the proportion of time wet, in 10-min blocks [31]. Devices were deployed for single foraging trips, after which birds were recaptured and the loggers removed. Logger deployment did not cause any observed injury, distress or adverse changes in behavior.

Here, we analyze only direct, sustained, bouts of flight, which we define as those during which straightness was ≥0.8 and the proportion of time on the water was < 0.5. Following [8], straightness for the *i*th location *L*_,_ is the straight line distance between locations *L*_i-1_ and *L*_i + 2_ divided by the along track distance between these locations. Ground velocity between locations was then calculated, correcting for time actually spent in flight.

We obtained wind data, consisting of 6-hourly zonal and meridional wind speed components at a nominal height of 10 m above sea level, from the European Center for Medium-Range Weather Forecasts (ECMWF) on a reduced Gaussian grid (minimum node spacing at Bird Island, South Georgia (54°S),125 km in latitude × 75 km in longitude). These data were produced by assimilation and reanalysis of observations of the global atmosphere, including wind speeds measured using the SeaWinds scatterometer aboard the QuikSCAT satellite, and observations from marine and terrestrial platforms [32]. The data are published as ERA-Interim dataset, available for download at http://apps.ecmwf.int/datasets/data/interim-full-daily/levtype=sfc/. We identified wind speed estimates nearest in time to each bird location and estimated wind speeds at these locations by linear interpolation between the two most spatially proximate grid points. We then reduced wind speeds to a reference height of 5 m above mean sea level (the median flight height for albatrosses observed from Bird Island) [9], assuming a logarithmic average wind profile and a scale height of 0.03 [9, 13].

### Statistical methods

Animal tracking data result from observing individuals repeatedly, typically leading to within-individual and serial autocorrelation [33]. To account for these sources of autocorrelation, and thereby draw robust statistical inferences, we used linear mixed-effects models (LMMs) to investigate the dependence of albatross flight speed on the speed and relative direction of the wind, treating individual as a random effect. All models were fitted in the R package nlme [34]. Each includes bird-level random intercepts (to account within-individual autocorrelation) and a first order continuous autoregressive term to model serial autocorrelation [8, 35]. We also included sex as a fixed effect, as this has previously been shown to affect albatross flight speed [8]. We checked the assumptions that residuals were normally distributed and homoscedastic using normal Q-Q plots and plots of fits vs. residuals. In order to estimate the variance explained by the fixed effects in each model, we calculated Nakagawa and Shinichi’s marginal *R*^2^_LMM(m)_ [36]. Unless otherwise stated, data are presented as means ± standard errors or medians with inter-quartile ranges (IQRs).

### Leeway model

The speed and direction of each bird over the ground, between each pair of locations (ground velocity) was calculated by dividing the distance by the time of flight between those locations. We also calculated the relative direction of ground velocity with respect to wind velocity. We assumed symmetry in albatross ground speeds in the across-wind direction (i.e. mean speed is the same, regardless of whether the birds are flying to the right or left of the wind direction) and therefore combined all observations into directions from zero (downwind) to 180° (upwind) relative to the wind direction.

**Fig. 2:**
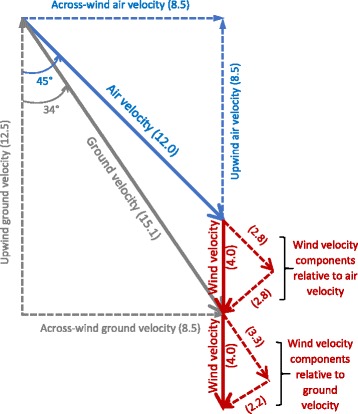
Vectoral decomposition of wind, air and ground velocities. Unbroken arrows indicate true vectors, while dashed lines indicate vectoral components parallel air and ground velocity. All speeds are in m/s. In this example, a 4.0 m/s uniform wind blowing toward the bottom of the figure (southward) would advect a bird downwind at 4 m/s as it flies at 12.0 m/s on a heading of 45 degrees relative to the downwind direction. The bird’s ground velocity (velocity over the ground) is the vector sum of air velocity (velocity of the bird through the air) and wind velocity. The resulting ground velocity would be 15.1 m/s at an angle of 34 degrees relative to the wind velocity. If both the ground velocity of a bird and the wind velocity were measured one could calculate the bird’s air velocity by subtracting wind velocity from ground velocity. Because an albatross soars through the wind-shear boundary layer, estimating downwind advection velocity is more complicated than simply using the wind velocity at the average height of the bird (see Methods). We refer to the downwind advection velocity by the wind as “leeway velocity,” which we estimate from the wind and flight data. In this study leeway velocity is around one half of the wind velocity at our chosen reference height of 5 m.downwinddownwinddownwind

We define leeway velocity as the downwind advection velocity of a bird by wind velocity (Fig. 2), which tends to increase downwind ground velocity and decrease upwind ground velocity. In the case of dynamic soaring birds, leeway velocity is usually different from wind velocity at typical assumed reference heights because of the vertical shear of wind velocity near the ocean surface. Rather, leeway velocity is equal to the average wind velocity encountered by a bird as it soars vertically through this boundary layer.

In order to estimate leeway velocity, we assumed that it is proportional to wind velocity and can be calculated from the observed variations of ground velocity associated with variations of wind velocity. This same assumption was used as a basis to calculate fine-scale estimates of wind velocity from high resolution GPS measurements of the dynamic soaring maneuvers of seabirds [37]. Specifically, we modeled ground speed (magnitude of ground velocity) as a linear function of the component of wind velocity in the direction of ground velocity, *W*cos*θ*, where *W* is the wind speed at a reference height of 5 m (the median height of albatrosses observed near Bird Island) and *θ* is the relative angle between wind velocity and ground velocity [8].

1 $$ \mathrm{Ground}\ \mathrm{speed}\ \left(\mathrm{m}/\mathrm{s}\right)={\alpha}_s+\beta Wcos\theta . $$

The intercept *α*_*s*_ (subscript *s* refers to sex) estimates the mean ground speed of males or females and the slope parameter *β* is the fraction of the wind velocity causing leeway. This formulation implicitly assumes that ground speed does not vary with wind speed or the relative direction of the wind except through the second term, which represents leeway. We calculated leeway velocities as being equal to the slope parameter *β* times wind velocities, as given in Eq. 2 below, where *β* was determined with (Eq. 1)

2 $$ \mathrm{Leeway}\ \mathrm{velocity}=\beta \left(\mathrm{wind}\ \mathrm{velocity}\right). $$

Ground velocity is then the vector sum of air velocity and leeway velocity (Fig. 2), which means that we can calculate air velocities by subtracting leeway velocities from GPS-derived ground velocities as in Eq. 3

3 $$ \mathrm{Air}\ \mathrm{velocity}=\mathrm{Ground}\ \mathrm{velocity}\hbox{-} \beta \left(\mathrm{wind}\ \mathrm{velocity}\right). $$

We also calculated the relative direction over this period (i.e. the orientation of the bird with respect to the downwind direction).

We evaluated the vector subtraction method used to estimate air velocity (Eq. 3) with the help of numerical simulations and assumed distributions of airspeeds relative to the wind direction, including several different assumed wind speeds and a typical increase of airspeed with increasing wind speed. We calculated leeway velocities using *β* = 0.5 times wind velocities (see Results) and then ground velocities by adding leeway velocities to the assumed air velocities. Using these values, we calculated the slope *β* relating ground speed to the component of wind in the direction of ground velocity (Eq. 1) to be within around 4% of the assumed *β* = 0.5, varying somewhat depending on the particular combination of assumed airspeeds and wind speeds. This demonstrated that our method correctly obtained an appropriate value of the slope parameter *β*.

It should be cautioned that estimated leeway velocities could include an unknown error resulting from variations of ground speed due to variations of the along-wind component of air velocity associated with variations in wind velocity. Since downwind components of air velocity were calculated by subtracting estimated leeway velocities from downwind components of ground velocity, there is uncertainty in our estimate of airspeed and the resulting airspeed polar diagram. Direct measurements of airspeed would be required to estimate this error. However, the ground speed polar diagram would not be affected because estimated leeway velocities were added back to air velocities in order to obtain modeled ground velocities.

### Airspeed model

Exploratory analyses of the data indicated that the form of the relationship between albatross airspeed (magnitude of air velocity) and relative wind direction is approximately sinusoidal, with maximum airspeeds (~ 20 m/s) occurring in the across-wind direction. Simple linear models indicate that airspeed generally increases with wind speed but the intercept and slope of this relationship varies with the relative wind direction. Taking these trends into consideration, we modeled airspeed as

4 $$ \mathrm{Airspeed}={a}_s+{\beta}_1W+{\beta}_2 Wsin\theta +{\beta}_3 W\theta +{\beta}_4\theta . $$

and used LMMs to estimate the coefficients *α*_*s*_ and *β*_*1*_-*β*_*4*_, where *θ* is the angle between wind velocity and a bird’s air velocity. The first term *α*_*s*_ is the intercept for males or females. The next two terms model variations in airspeed symmetrically in the upwind and downwind directions, where *W* is the wind speed and *W*sin(*θ*) is the component of wind velocity perpendicular to air velocity. The fourth and fifth terms model variations in airspeed as a function of *θ* that are asymmetric in the upwind/downwind directions.

### Ground speed model

We calculated modeled ground velocities (Eq. 5) by adding leeway velocities (*β*(wind velocities) Eq. 2) to modeled air velocities (Eq. 4)

5 $$ \mathrm{Modeled}\ \mathrm{ground}\ \mathrm{velocity}=\mathrm{Modeled}\ \mathrm{air}\ \mathrm{velocity}+\beta \left(\mathrm{wind}\ \mathrm{velocity}\right). $$

where modeled air velocity is defined by the modeled airspeed and the associated relative wind direction used in Eq. 4, and wind velocity is defined as the associated wind speed in the downwind direction.

## Results

### Tracking data

Of 8060 tracking locations recorded from wandering albatrosses, 11% (*n* = 884) met the selection criteria and were therefore retained for analysis. The median proportion of time spent on the water by birds between these locations was 0.05 (IQR 0.00–0.12), indicating that the vast majority of data were from nearly unbroken bouts of flight. Median straightness during these bouts was 0.90 (IQR 0.85–0.96).

### Leeway

**Fig. 3:**
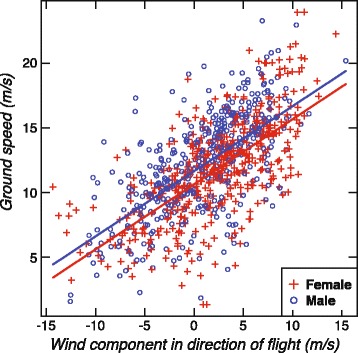
Ground speed (magnitude of ground velocity) of wandering albatrosses measured by GPS, modeled as a function of sex and the component of wind velocity in the direction of flight (*W*cos*θ*), where *W* is wind speed and *θ* is the relative angle between wind velocity and ground velocity (*θ* = 0° is downwind). Data are from 22 female birds (479 locations) and 24 male birds (404 locations). The intercepts (females 10.6 m/s, males 11.6 m/s) represent the average ground speed in across-wind flight. The slope of the lines is 0.51 and indicates the fraction of wind velocity that is equal to leeway velocity (Eq. 1)

Figure 3 shows ground speeds (magnitudes of ground velocities) plotted as a function of the components of wind velocity in the direction of ground velocities, and Table 1 gives our estimates of the parameters in Eq. 1. The model provides a reasonable fit to the data (*R*^2^_LMM(m)_ = 0.43, 95% CIs 0.39–0.48). The mean ground speed of males is 0.97 (±0.27) m/s faster than that of females, and the slope parameter *β* is 0.51 (±0.02). The effective leeway velocity is approximately one half of the wind velocity at 5 m height (Table 1). This is probably because although albatrosses soar in the wind-shear boundary layer, they spend considerable amounts of time below a height of 5 m and in wave troughs shielded from the full force of the wind.

**Table 1:** Wandering albatross ground speed (m/s) as a function of wind speed and sex (Eq. 1)

	Value	SE	df†	*T*	*p*
*α*_*f*_	10.59	0.20	836	53.34	< 0.001
*β*	0.51	0.02	836	23.22	< 0.001
*α*_*m*_ *- α*_*f*_	0.97	0.27	44	3.39	0.002

† Estimated degrees of freedom

*α* = mean ground speed of males (*α*_*m*_) and females (*α*_*f*_), respectively (m/s)

*β* = slope of line relating ground speed and the component of wind velocity in the direction of flight (ground velocity)

### Airspeed

**Fig. 4:**
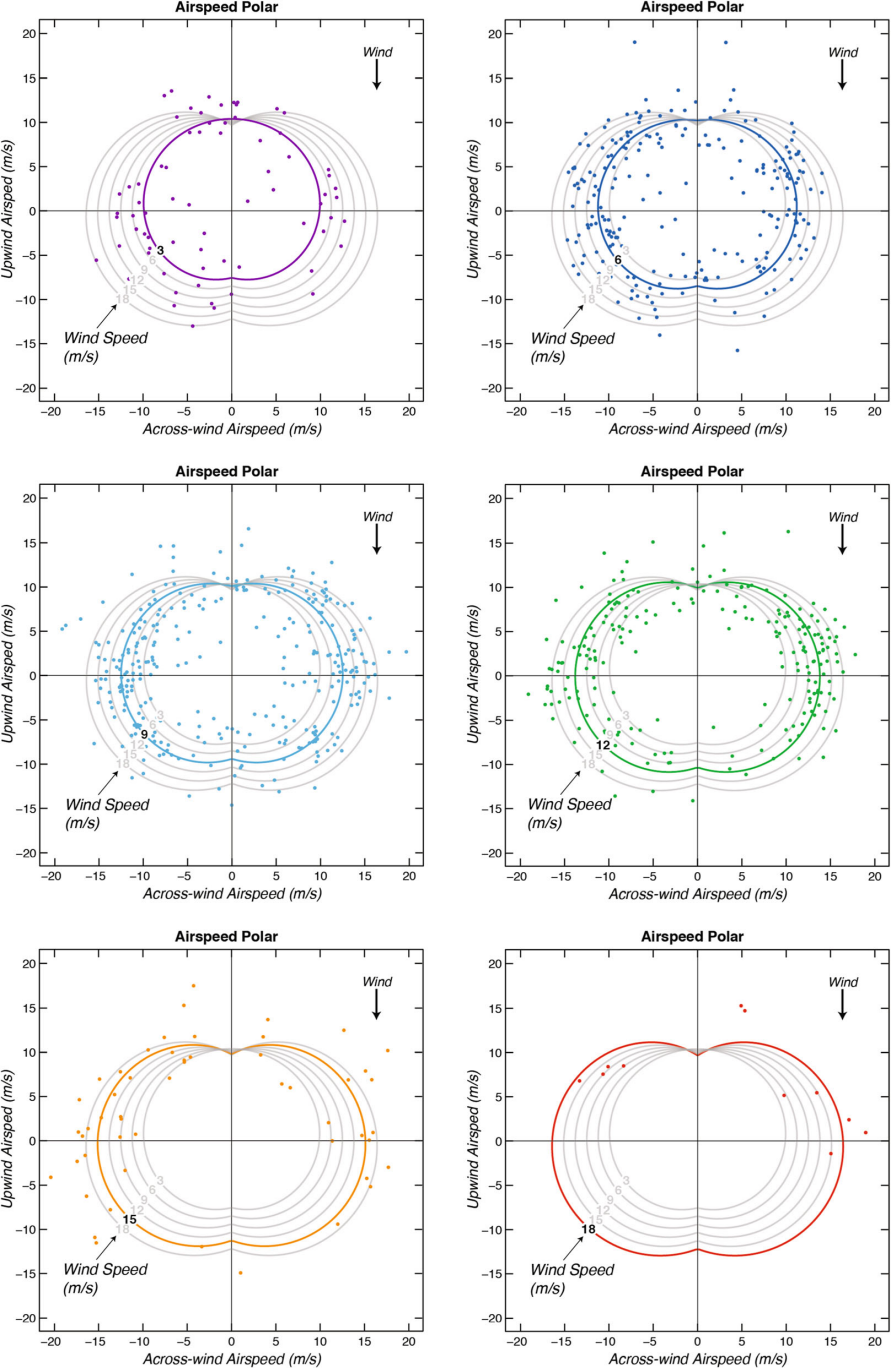
Polar diagrams showing estimates of wandering albatross airspeed (magnitude of flight velocity through the air or air velocity) with flight direction relative to the wind (colored dots). Each curve represents modeled airspeed averaged across the sexes as a function of relative wind direction for a specific wind speed as calculated with a linear model (Eq. 4). Colored airspeed values are associated with wind speeds within 1.5 m/s of the wind speed of each similarly colored model airspeed curve. The average wind speed is 9.0 m/s so the airspeed curve for this wind speed (light blue) is representative of average airspeeds. Across-wind components of air velocity are plotted against upwind components of air velocity

Airspeed values (magnitudes of air velocities) plotted against relative wind direction (Fig. 4) indicate that maximum airspeeds tend to lie in the across-wind direction. The average of all airspeeds is 11.9 (±0.1) m/s, and the average wind speed is 9.0 (±0.1) m/s. The average of the nine fastest airspeeds (fastest 1%) in the across-wind direction (45–135 degrees) is 19.7 (± 0.2) m/s.

### Dependence of airspeed on sex and wind speed

There is considerable variability in airspeed at different wind speeds and relative directions (Fig. 4), and the model described by Eq. 4 explained only about a quarter of the variation in airspeed (*R*^2^_LMM(m)_ = 0.26, 95% CIs 0.22–0.31). Nonetheless, all terms were all highly significant (Table 2), confirming that airspeed is dependent on sex, wind speed and flight direction relative to wind direction. The mean airspeed of males is 0.88 (±0.24) m/s faster than that of females.

**Table 2:** Wandering albatross airspeed (m/s) as a function of wind speed, relative wind direction, and sex (Eq. 4)

	Value	SE	df†	*T*	p
*α*_*f*_	6.18	0.71	833	8.66	< 0.001
*α*_*m*_ *- α*_*f*_	0.88	0.24	44	3.65	0.001
*β*_*1*_ *(W)*	0.31	0.09	833	3.43	0.001
*β*_*2*_*(W*sin*(θ))*	0.30	0.04	833	8.38	< 0.001
*β*_*3*_ *(Wθ)*	−0.11	0.04	833	−2.88	0.004
*β*_*4*_ *(θ)*	1.26	0.35	8.33	3.61	< 0.001

† Estimated degrees of freedom

*α* = mean airspeed of males and females, respectively (m/s)

*β* = regression coefficients for the specified terms in parentheses

*W* = wind speed values (m/s)

*θ* = angles (radians) between wind velocities and air velocities

**Fig. 5:**
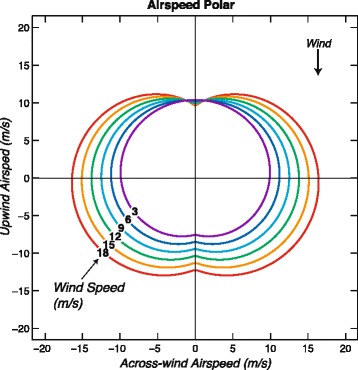
Polar diagram showing modeled airspeed averaged across the sexes as a function of wind speed and relative wind direction for six different wind speeds as calculated with a linear model (Eq. 4). The average wind speed is 9.0 m/s so the airspeed curve for this wind speed (light blue) is representative of average airspeeds. Across-wind components of modeled air velocity are plotted against upwind components of modeled air velocity

The spacing between modeled airspeed curves in Fig. 5 clearly illustrates that across-wind airspeeds increase with wind speed faster than do upwind and downwind airspeeds. The upwind airspeed is nearly constant as a function of wind speed and at low wind speeds is larger than downwind airspeed. At higher wind speeds the airspeed polar is oval-shaped with maximum airspeeds occurring in the across-wind direction.

Equations for airspeed of females in the downwind (0°), across-wind (90°) and upwind (180°) directions determined from Eq. 4 are as follows:

4a $$ \mathrm{Downwind}\ \mathrm{airspeed}\ \left(m/s\right)=6.2+0.31W. $$

4b $$ \mathrm{Across}\hbox{-} \mathrm{wind}\ \mathrm{airspeed}\ \left(m/s\right)=8.2+0.44W. $$

4c $$ \mathrm{Upwind}\ \mathrm{airspeed}\ \left(m/s\right)=10.1-0.04W. $$

As a confirmation of Eq. 4b, we also modeled across-wind airspeed values (relative directions 45–135°) as a function of sex and wind speed. Results for females are an intercept of 7.9 (±0.4) m/s and slope of 0.42 (±0.04), which are in close agreement with Eq. 4b. The intercept for males is 0.88 m/s greater than that for females. These values indicate an increase of airspeed of around 6 m/s over the 15 m/s increase of wind speed values (3 to 18 m/s). This confirms Hypothesis 1 (that airspeed increases with wind speed), with the caveats that this effect occurs in all relative directions other than directly upwind and that it is most marked in flight across the wind (Fig. 5, Table 2). The relationship between across-wind airspeeds and wind speed implies that average airspeeds continue to increase up to at least *W* = 20 m/s. However, the fastest measured across-wind airspeeds appear to plateau at around 20 m/s for wind speeds above around 8 m/s, continuing at this level up to at least *W* = 20 m/s (Fig. 4). This suggests that wandering albatrosses can fly increasingly fast across-wind with increasing winds until reaching a maximum airspeed at around 20 m/s. The plateau of values near 20 m/s indicates the 0.42 slope parameter ceases to be valid for fast wind speeds.

Numerous airspeed observations are considerably faster than the mean predicted airspeed curves in Fig. 4. These fast airspeeds represent a measure of the upper limit of airspeed performance of the birds. The average of the fastest 10% of the residuals about Eq. 4 is 4.5 (± 0.1) m/s. This suggests that wandering albatrosses can at times fly around 4.5 m/s faster than average airspeeds. Fast airspeeds can be modeled by adding 4.5 m/s to the intercepts in Eq. 4.

**Fig. 6:**
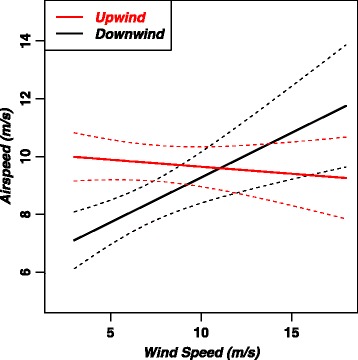
Mean airspeed (magnitude of air velocity) of female wandering albatrosses in upwind and downwind flight predicted as a function of wind speed. Dashed lines show 95% confidence intervals

At wind speeds less than around 11 m/s, upwind airspeed is greater than downwind airspeed (Fig. 5 and Eqs. 4a and 4c), the difference being greatest at the slowest wind speeds. The *β*_*4*_*θ* term in Eq. 4 indicates that upwind airspeed is larger than downwind airspeed by 4.0 (± 1.1) m/s for *W* = 0, although this airspeed is below the minimum 3–4 m/s required to support dynamic soaring. The difference in airspeeds for *W* = 3 m/s is 2.9 (± 1.2), this partially confirms Hypothesis 2 that albatrosses increase airspeed in upwind flight relative to that in downwind flight, but this effect is only significant at low wind speeds (< ~ 7 m/s) and is reversed (but not significant) at higher wind speeds (Fig. 6).

### Ground speed

**Fig. 7:**
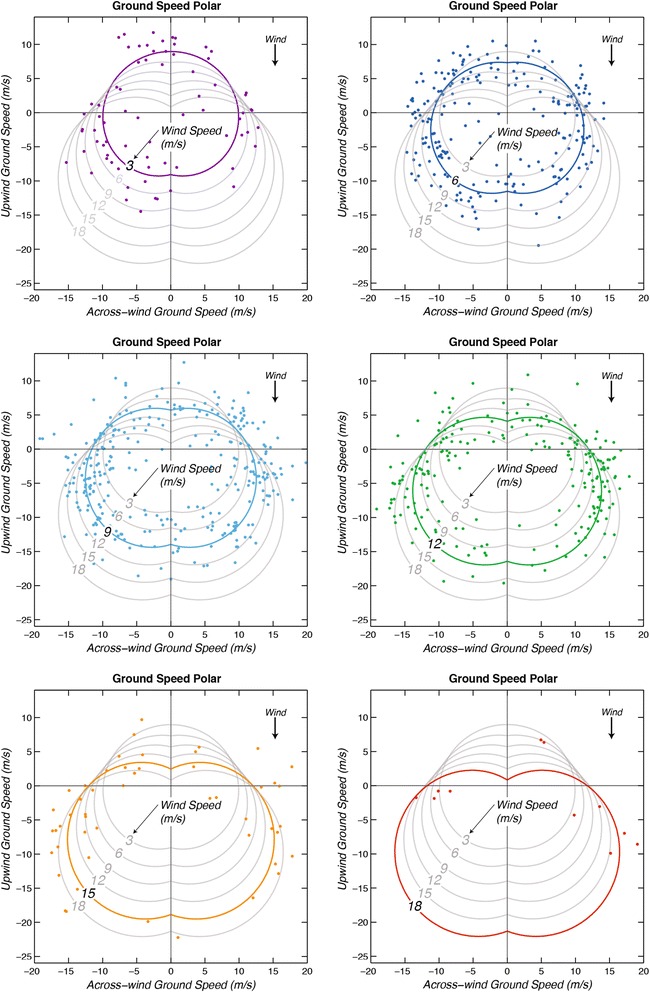
Polar diagrams showing observations of wandering albatross ground speed (magnitude of ground velocity) and flight direction relative to the wind direction (colored dots). Curves show modeled ground speed as a function of relative wind direction for six different wind speeds (Eq. 5) calculated by adding leeway velocity (0.51 times wind velocity, Eq. 2) to modeled air velocity (Eq. 4). Colored ground speed values are associated with wind speeds located within 1.5 m/s of the wind speed of each similarly colored model ground-speed curve. The modeled ground-speed curve for 9.0 m/s wind speed (light blue) is representative of average ground speeds. Two fast outliers near an upwind ground speed of 16 m/s fall above the top of the plot and are not shown. Across-wind components of ground velocity are plotted against upwind components of ground velocity

**Fig. 8:**
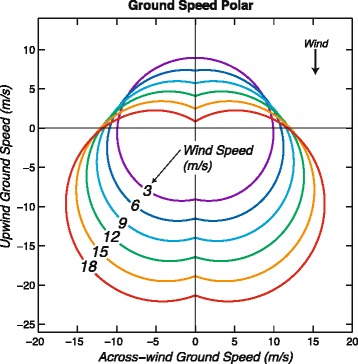
Polar diagram showing curves of modeled ground speed (magnitude of ground velocity) as a function of relative wind direction for six different wind speeds (Eq. 5) calculated by adding estimated leeway velocity (0.51 times wind velocity, Eq. 2) to modeled air velocity (Eq. 4). The modeled ground-speed curve for 9.0 m/s wind speed (light blue) is representative of average ground speeds. Across-wind components of modeled ground velocity are plotted against upwind components of modeled ground velocity

Observed and modeled ground speeds are shown in Figs. 7 and 8 in the form of polar diagrams. The average ground speed is 12.0 (± 0.1) m/s. The average wind speed is 9.0 m/s, so the light blue curve for that wind speed is representative of average ground speeds. Notably, due to the combination of fast airspeeds and leeway the fastest ground speeds (~ 22 m/s) tend to be located in the diagonal downwind direction.

Modeled ground speed curves (Fig. 8) calculated using Eq. 5 for specific wind speeds have the same shape as their equivalent airspeed curves (Fig. 5) but are displaced downwind by leeway. This indicates that a major source of variability in ground speeds is due to leeway. Several curves cross each other near the intersections with the line representing the zero-upwind ground speed, explaining the numerous measured ground speed values grouped in these regions (Figs. 7 and 8). This is because increasing airspeed is partially countered by increasing leeway in these parts of the polar.

## Discussion

### Airspeed

The difference in airspeed between male and female wandering albatrosses corresponds in magnitude to the between-sex difference in best glide speed predicted using an aerodynamic model [8], and is likely to be due to the significantly higher wing loading of males [38]. Our data support the hypotheses both that albatross airspeed is dependent on wind speed and that albatrosses increase airspeed in upwind flight relative to that in downwind flight. However, this only occurred at low (< ~ 7 m/s) wind speeds. The latter is consistent with the prediction that the optimal range speed of birds is higher in headwind than tailwind flight [24, 25]. There is some debate about whether this hypothesis applies to soaring birds, such as albatrosses, because it has been assumed that energy expenditure during this mode of flight is independent of flight direction with respect to wind [10]. However, the energy expenditure of free-ranging wandering albatrosses (inferred from heart rate) varies as a function of flight direction relative to the wind, peaking in headwind flight [28]. This could be because greater forces are applied to the wings in upwind flight, necessitating more energy to maintain flight posture, or because flight maneuvers requiring muscular adjustments are more frequent in upwind flight. Regardless of the mechanism, our results suggest that airspeed optimization occurs in albatrosses and might well reflect energetic considerations. An alternative, non-exclusive, explanation for the observed variation in airspeed is that this occurs to optimize energy gain during foraging [10]. However, this seems unlikely as our analyses focused on periods when birds were in direct flight, during which they would mainly have been commuting between the colony and distant prey patches, rather than actively foraging.

The across-wind airspeed of the birds reached a maximum of ~ 20 m/s (Fig. 4). This implies that they limit their airspeeds at higher wind speeds, probably to keep the force on their wings encountered in dynamic soaring well below the limit of wing strength. Possible ways a bird could do this are by adjusting the shape of its wings, by decreasing the frequency of shear-layer crossings, by reducing the amount of height gained when climbing through the wind-shear layer, and by remaining above the region of strongest wind-shear.

The average increase of across-wind airspeed as a function of wind speed was found to be 0.44 *W* (Eq. 4b). This value is only about a tenth of the maximum possible predicted using a two-layer model optimized for maximum airspeed of a wandering albatross [39]. Optimized fast airspeed as a function of wind speed requires an increase of the frequency of dynamic soaring maneuvers, which can result in accelerations that are too large to be supported by the wings. Thus, in principle, dynamic soaring over the ocean could provide sufficient energy for possibly much faster flight of strong robotic “albatross-like” UAVs.

### Ground speed

The dominant variation of ground speed is ~ 8 m/s in the along-wind direction due to leeway (Fig. 8). However, there are ranges of wind speeds and relative directions in which the variations of airspeed as a function of wind are larger than the variation of ground speed due to leeway. For example, in across-wind flight the average increase of airspeed (and ground speed) with wind speed is around 6 m/s over the 15 m/s range of wind speeds from 3 to 18 m/s (Fig. 5). There is also a series of diagonally upwind airspeeds, which have the same magnitude as the corresponding series of diagonal downwind ground speeds. For these series the increase of airspeed with increasing wind speed is dominant. Therefore, variations of airspeed due to variations of wind speed can be substantial and need to be incorporated in models of albatross flight, especially in the across-wind direction.

Maximizing upwind ground speed requires both maximizing upwind airspeed and minimizing leeway speed. However, these two goals require somewhat different flight maneuvers. Maximizing upwind airspeed requires full use of wind shear to gain as much energy as possible (within limits imposed by the structural strength of the wings) and fast airspeeds to soar upwind against contrary winds and waves. Minimizing leeway speed requires a reduction in time spent in fast contrary winds and an increase in time spent in regions of low wind speed and in wave troughs. Although local wind waves generally propagate downwind, often one sees a combination of wind waves and swell waves propagating from elsewhere at an angle to the wind waves. An albatross might be able to exploit resulting wave troughs to reduce leeway [39]. Because of the complexity involved in maximizing upwind airspeed at the same time as minimizing leeway speed, an albatross has to work to soar fast upwind. An indication of the difficulty of upwind flight is the near-zero increase of upwind airspeed and the decreasing upwind ground speeds with increasing wind speed. The effort to soar upwind has been documented by measurements of increased heart rates of wandering albatrosses soaring upwind [28] compared to soaring across-wind or downwind where leeway is not a hindrance and can be helpful in increasing ground speed. It is possible that the increased energetic demands of upwind flight are due to the increased frequency and complexity of flight maneuvers required compared to across-wind or downwind flight since wandering albatrosses infrequently flap their wings while soaring upwind (or in any other direction) as long as there is sufficient wind for dynamic soaring [9, 39].

Some implications for optimal flight strategies can be inferred from the ground speed polar (Fig. 8). During flight directly upwind (*θ* = 180°), ground speeds decrease with increasing wind speed from around 9 m/s at *W* = 3 m/s to around 1 m/s at *W* = 18 m/s. This is a result of nearly constant airspeeds in this direction (Fig. 5) minus increasing leeway ~ 0.51 *W*, which amounts to a decrease in ground speed of around 7.5 m/s over wind speeds of 3–18 m/s. The curves in Fig. 8 clearly illustrate that upwind ground speeds are much faster in low wind speeds than in faster wind speeds. Albatrosses tend to resort to upwind flight infrequently, presumably due to its energetic cost [8, 28] but under some circumstances it may be necessary. For example, birds may need to progress rapidly upwind in order to exploit ephemeral prey patches or to return to the colony to provision offspring. Under these circumstances, and at wind speeds greater than around 9 m/s, it would be slightly faster to make headway directly upwind by tacking diagonally upwind like a sailboat. High resolution GPS-tracking [40] shows that wandering albatrosses approaching prey upwind indeed tack from side to side with an across-wind horizontal scale of ~ 0.5 km. This has been attributed to olfactory searching [40], but it may also increase the speed with which albatrosses close on prey detected by smell. As more seabirds are tracked using high-resolution devices it will become apparent whether tacking is a movement strategy employed widely among albatrosses and other dynamic soarers. For example, a high resolution GPS track of a wandering albatross obtained by Geoff Bower (personal communication, 2014) documented a nearly-straight (average) direct upwind flight.

Our models predict that in wind speeds of 18 m/s, the maximum upwind ground speed of a wandering albatross would be at an angle of around 65° relative to the upwind direction. At a wind speed of around 25 m/s the average upwind ground velocity would be zero even for diagonal upwind flight (Eq. 5). However, some measured upwind ground speeds are around 4–5 m/s faster than the curves in Fig. 5. This suggests either that some birds could make greater headway upwind than our model suggests (Fig. 5), or that wind speeds and directions (which were measured only every 6 h) were in error, as described below for two obvious outliers. The relatively slow upwind flight speeds in fast winds imply that in order to make rapid progress in an upwind direction, albatrosses could also divert laterally around the region of fast contrary winds and low-pressure systems to exploit more favorable wind patterns, such as lower wind speeds and better relative wind direction. Some examples of large-scale loops around strong contrary winds have been shown for *Diomedea* spp. [28, 41]. We caution that our dataset includes few observed airspeeds at wind speeds above 15 m/s and only one above 20 m/s, which means that results inferred from Eq. 5 for fast wind speeds (> 20 m/s) are based on extrapolations with a large uncertainty. Clearly, additional tracking data in high winds are needed in order to improve polar diagrams and better define flight patterns of higher airspeeds and ground speeds.

High resolution trajectories of wandering albatrosses obtained with GPS measurements have helped to resolve details of typical dynamic soaring maneuvers [4, 27]. Two examples of average across-wind trajectories indicate a series of linked ~ 90° turns consisting of a climb at a relative angle to the wind of around 135°, a 90° downwind turn, a descent at an angle of around 45°, and a 90° upwind turn. In one of these examples average across-wind ground velocity is 16.3 m/s (*θ* ~ 65°) in an average wind speed (estimated for 5 m height) of 6.9 m/s. In the second example average across-wind ground velocity is 15.5 m/s (*θ* ~ 86°) in an average wind speed (5 m height) of 14.9 m/s. The average period of two 90° turns was around 10 s for both examples. The average across-wind ground velocity for these two examples is around 3 m/s faster than the values predicted using the GPS measurements analyzed here (Eq. 5) but not as fast as the fastest measured ground speeds in Fig. 7. Two examples of upwind ground speeds [27] are around 2 m/s faster than those predicted with Eq. 5. These examples tend to confirm that the fast commuting ground speeds estimated with GPS positions at intervals of 30+ minutes are realistic.

The ground speed polar diagrams (Fig. 7) have some similarities to an earlier one [10]. The new diagrams indicate ground speeds that are around 2–4 m/s faster. This could reflect data from faster individuals, particularly given the much larger number of observations (883 vs. 57) in the present study. The earlier study has a reduced scatter of ground speeds as plotted against relative direction, presumably because of the shorter averages of the optical measurements compared to the GPS data. It would be interesting to use high-resolution data with in-situ wind measurements to calculate an effective leeway speed, an airspeed polar diagram, and to compare airspeeds and the increase of airspeed with wind speed with the values found for wandering albatrosses.

### Model criticism

While all terms in both our model of airspeed and ground speed were highly significant (Tables 1 and 2), neither model explained more than 50% of the variation in the observed phenomena. Here we discuss a number of potential reasons for the unexplained variability. Firstly, variation may have arisen due to spatiotemporal mismatches in the wind and tracking data and due to errors in the wind speed estimates. Wind data were only available at a relatively coarse resolution (~ 100 km, 6 h). Secondly, the assumed vertical wind profile used to obtain the wind at 5 m could differ from the profile encountered by the birds due to variation in sea state. Thirdly, wind speed estimates based on scatterometer data may be biased downwards compared to true wind speeds measured by ocean buoys. In our analysis, this would lead to error in the interpreted variation in airspeed, potentially biasing our results. However, this effect is thought to be relatively small, especially in light winds (satellite estimates ~ − 0.12 times true wind speeds [42]). Due to the relatively long interval between tracking locations in our study (0.5–2 h), errors in speed estimates due to GPS error would be negligible (≤ 20 m error in distance, over the typical distances of ~ 40 km between GPS positions). However, some slower observed ground speeds could have resulted from deviations from a straight track during the intervals between GPS positions. Wandering albatrosses have been observed to fly in various complicated flight maneuvers at fine scales of around 100 m associated with dynamic soaring, and also at larger scales as the birds search for food and respond to varying winds [43]. This might explain why even though we restricted our analysis to track segments with a straightness of > 0.8 some across-wind examples of high resolution GPS tracks [27] were 3 m/s faster than the average trends modeled here. Similarly, our analysis included bouts of movement during which birds spent some time on the water. However, we calculated speed based on time in flight and the median proportion of time spent on the water by birds in the dataset analyzed was 0.05 (IQR 0.00–0.12), suggesting that this would have introduced little bias. Ultimately, higher resolution tracking data would resolve finer scale changes in the behavior, ground speed and airspeed of albatrosses, thereby providing more accurate insights into the aerodynamics of albatross flight, e.g. [4, 27, 40]. Finally, some variation in predicted speeds may be due to the different wing loadings of individual birds, as exemplified by the significant difference of ~ 1 m/s between males and females (Table 2, Fig. 3). In future studies, measuring the wing profiles and masses of study birds, and thereby deriving their wing loading, could help to explain this variation.

Given these limitations, it should be cautioned that airspeeds predicted by our models will almost certainly be less than instantaneous values of airspeed experienced by the bird in dynamic soaring maneuvers. For example, if an albatross were flying across-wind in an S-shaped series of 90° linked curves the airspeed calculated from GPS fixes as described above would be approximately 90% of the average airspeed of the bird along its soaring trajectory (Fig. 1). Therefore, it is problematic to compare an average airspeed determined from GPS positions with the ~ 16 m/s cruise airspeed coinciding with the maximum glide ratio and the ~ 11.5 m/s airspeed coinciding with the minimum sink rate of a wandering albatross in straight flight [7] unless the details of the dynamic soaring maneuver are measured.

### Implications for dynamic soaring and foraging

Visual observations and model simulations of a wandering albatross that was soaring upwind in ~ 7 m/s winds with upwind ground speeds of around 12 m/s [39] are similar to some faster values documented here by GPS tracking. Since details of dynamic soaring flight maneuvers at fine temporal scales are lacking in our GPS data, the visual observations are offered as a possible explanation of how the tracked albatrosses accomplished upwind flight.

The observed upwind flight consisted of a series of ~ 90° turns. Starting with across-wind flight in a wave trough, the bird turned ~ 90° toward the wind direction and climbed upwind across the wind-shear layer, followed by a ~ 90° turn toward an across-wind direction and a descent into another wave trough. The period of the maneuver was around 10 s. By alternating directions of ~ 90° turns a bird can progress diagonally upwind or even (on average) straight upwind like a sailboat tacking into the wind. Some high-resolution GPS tracks have been published [4, 27, 40], but the trajectories as seen on the small figures are complicated and difficult to interpret quantitatively. There appear to be examples of both diagonal upwind flight and direct upwind flight by tacking. Further analysis of high temporal resolution tracks could provide details of fine-scale dynamic soaring flight maneuvers, estimates of fine-scale wind velocities [37], and more accurate airspeed and ground speed polars.

The oval-shaped airspeed polar diagram (Fig. 5) can be explained by considering the mean air velocities associated with the linked series of 90° turns for both across-wind and diagonal upwind flight. At the minimum wind speed needed to support dynamic soaring, a wandering albatross can soar at its 16 m/s cruise airspeed, which is associated with the maximum glide ratio of 21.2 [7]. Assuming an average airspeed of 16 m/s along a bird’s dynamic soaring trajectory, the mean across-wind air velocity would be around 14.4 m/s, as would the diagonal upwind and diagonal downwind air velocities. In increasing wind speeds a bird can increase its mean air velocity (Fig. 5). More airspeed and kinetic energy can be obtained (per unit time) from crossing the wind-shear layer in across-wind flight than in diagonal upwind or diagonal downwind flight, possibly explaining why the fastest mean air velocities are located in the across-wind direction.

A two-layer model of wind speed consisting of zero wind in the lower layer and a uniform wind *W* in the upper layer helps in estimating the increase of airspeed and kinetic energy when a bird crosses the wind-shear layer [39]. Diagonal upwind flight gains an airspeed equal to ~ *W* during the directly upwind climb across the wind-shear layer associated with the two 90° turns (in 10 s), the descent being in an across-wind direction with little airspeed gain or loss. Across-wind flight gains airspeed equal to ~ 0.7 *W* on both the diagonal upwind and diagonal downwind crossings of the wind-shear layer, resulting in an increase of around 1.4 *W* in the two 90° turns (in 10 s), or around 40% more airspeed than that obtained in diagonal upwind flight. This larger gain in airspeed and kinetic energy could be used to soar faster in the across-wind direction than in the diagonal upwind or diagonal downwind directions, as observed (Fig. 4).

Fast across-wind airspeeds combined with leeway lead to the fastest ground speeds. In turn, this would increase the area that could be searched by a bird per unit time, thereby potentially increasing its foraging rate. For example, high resolution tracking has shown that wandering albatrosses soaring across wind turn upwind on average 2.5 km from sites of prey capture, the implication being that they detect prey by olfaction [40]. The success of this strategy is contingent not only on the bird’s ability to search rapidly during across-wind flight but to make headway in the final approach in upwind flight. Our results imply that in high winds (9–25 m/s) this could be achievable using a zigzag tacking maneuver.

## Conclusions

We analyzed wandering albatross flight velocities with respect to wind speeds estimated by reanalysis of satellite and direct observations of the global atmosphere, and modeled airspeed and ground speed as functions of wind speed and relative wind direction. In addition to providing new information about flight performance, our results could be used to model how the migration and foraging of albatrosses is facilitated or constrained by the wind field.

A major conclusion of our study is that wandering albatrosses can increase their across-wind airspeeds with increasing wind speeds by around 6 m/s, and can reach maximum across-wind airspeeds of around 20 m/s at wind speeds above 8 m/s (Fig. 4). The trend line through all across-wind airspeeds as a function of wind speed has a slope of 0.42 (±0.04). This results in an oval-shaped airspeed polar (Fig. 5) with fastest airspeeds centered in the across-wind direction. The birds probably limit maximum airspeed to around 20 m/s in order to ensure the aerodynamic force occurring in dynamic soaring is well below the level that would cause structural damage to their wings.

We modeled ground speed of wandering albatrosses using the speed and relative direction of the wind (Fig. 8). This has obvious application to analyses of movements relative to the wind field during migration, which can include circumpolar journeys of several tens of thousands of km in only 1–2 months [1]. We found ground speed to be dependent on three covariates: leeway velocity, the rate of increase of airspeed with wind speed, and the variation of airspeed with relative wind direction. Although leeway velocity (~ 0.5 *W*) dominates the variation of ground speeds in the along-wind direction (Fig. 8), variation of airspeed with wind speed (~ 0.4 *W*) dominates in the across-wind direction, which appears to be preferred by the birds (Figs. 4 and 5). This preference in relative direction is probably because a bird dynamic soaring in an across-wind direction can efficiently extract energy from wind shear while maintaining a fast average ground velocity over the ocean. Upwind ground speeds are around 9 m/s at low wind speeds (*W* ~ 3 m/s) but decrease as wind speeds increase to ~ 1 m/s at *W* ~ 18 m/s due to increasing leeway and nearly constant airspeeds in that direction (Fig. 8). Therefore, fastest upwind ground speeds tend to be achieved at low wind speeds. A small increase in upwind flight speed can be achieved in higher wind speeds by tacking diagonally upwind like a sailboat.

## References

[1] Weimerskirch H, Delord K, Guitteaud A, Phillips RA, Pinet P (2015). Extreme variation in migration strategies between and within wandering albatross populations during their sabbatical year, and their fitness consequences. Sci Rep.

[2] Rayleigh L (1883). The soaring of birds. Nature.

[3] Baines AC. The sailing flight of albatrosses. Nature. 1889;40:9-10.

[4] Sachs G, Traugott J, Nesterova AP, Bonadonna F (2013). Experimental verification of dynamic soaring in albatrosses. J Exp Biol.

[5] Delord K, Barbraud C, Bost C-A, Deceuninck B, Lefebvre T, Lutz R, Micol T, Phillips RA, Trathan PN, Weimerskirch H (2014). Areas of importance for seabirds tracked from French southern territories, and recommendations for conservation. Mar Policy.

[6] Froy H, Lewis S, Catry P, Bishop CM, Forster IP, Fukuda A, Higuchi H, Phalan B, Xavier JC, Nussey DH, Phillips RA (2015). Age-related variation in foraging behaviour in the wandering albatross at South Georgia: no evidence for senescence. PLoS One.

[7] Pennycuick CJ (2002). Gust soaring as a basis for the flight of petrels and albatrosses (Procellariiformes). Avian Science.

[8] Wakefield ED, Phillips RA, Matthiopoulos J, Fukuda A, Higuchi H, Marshall GJ, Trathan P (2009). Wind field and sex constrain the flight speeds of central place foraging albatrosses. Ecol Monogr.

[9] Pennycuick CJ (1982). The flight of petrels and albatrosses (Procellariiformes), observed in South Georgia and its vicinity. Philo Transaction of the Royal Society of London Series B-Biol Sci.

[10] Alerstam T, Gudmundsson GA, Larsson B (1993). Flight tracks and speeds of Antarctic and Atlantic seabirds - radar and optical measurements. Philo Transactions of the Royal Society of London Series B-Biol Sci.

[11] Lissaman P (2005). Wind energy extraction by birds and flight vehicles. Am Inst Aeronaut Astronaut Pap.

[12] Richardson PL (2011). How do albatrosses fly around the world without flapping their wings?. Prog Oceanogr.

[13] Sachs G (2005). Minimum shear wind strength required for dynamic soaring of albatrosses. Ibis.

[14] Idrac P (1925). Étude expérimentale et analytique du vol sans battements des oiseaux voiliers des mers australes, de l’Albatros en particulier. La Technique Aeronautique.

[15] Amélineau F, Péron C, Lescroël A, Authier M, Provost P, Grémillet D (2014). Windscape and tortuosity shape the flight costs of northern gannets. J Exp Biol.

[16] Ainley D, Porzig E, Zajanc D, Spear LB (2015). Seabird flight behavior and height in response to altered wind strength and direction. Mar Ornithol.

[17] Pistorius PA, Hindell MA, Tremblay Y, Rishworth GM (2015). Weathering a dynamic seascape: influences of wind and rain on a Seabird’s year-round activity budgets. PLoS One.

[18] Thorne LH, Conners MG, Hazen EL, Bograd SJ, Antolos M, Costa DP, Shaffer SA. Effects of el Niño-driven changes in wind patterns on North Pacific albatrosses. J R Soc Interface. 2016;13:20160196. 10.1098/rsif.2016.0196. 10.1098/rsif.2016.0196 PMC4938084 27278360

[19] Wynne-Edwards VC. On the habits and distribution of birds on the North Atlantic. Proceedings of the Boston Society of Nat History. 1935;40:233-346.

[20] Felicisimo AM, Munoz J, Gonzalez-Solis J (2008). Ocean surface winds drive dynamics of transoceanic aerial movements. PLoS One.

[21] Shaffer SA, Tremblay Y, Weimerskirch H, Scott D, Thompson DR, Sagar PM, Moller H, Taylor GA, Foley DG, Block BA, Costa DP (2006). Migratory shearwaters integrate oceanic resources across the Pacific Ocean in an endless summer. Proc Natl Acad Sci U S A.

[22] Weimerskirch H, Louzao M, de Grissac S, Delord K (2012). Changes in wind pattern alter albatross distribution and life-history traits. Science.

[23] Pardo D, Forcada J, Wood AG, Tuck GN, Ireland L, Pradel R, Croxall JP, Phillips RA (2017). Additive effects of climate and fisheries drive ongoing declines in multiple albatross species. Proc Natl Acad Sci.

[24] Pennycuick CJ (1978). 15 testable predictions about bird flight. Oikos.

[25] Hedenström A, Alerstam T (1995). Optimal flight speed of birds. Philo Transactions of the Royal Society of London Series B-Biological Sci.

[26] Pennycuick CJ, Croxall JP, Prince PA (1984). Scaling of foraging radius and growth-rate in petrels and albatrosses (Procellariiformes). Ornis Scand.

[27] Sachs G (2016). In-flight measurement of upwind dynamic soaring in albatrosses. Prog Oceanogr.

[28] Weimerskirch H, Guionnet T, Martin J, Shaffer SA, Costa DP (2000). Fast and fuel efficient? Optimal use of wind by flying albatrosses. Proceedings of the Royal Society of London Series B-Biol Sci.

[29] Fukuda A, Miwa K, Hirano E, Suzuki M, Higuchi H, Morishita E, Anderson DJ, Waugh SM, Phillips RA (2004). BGDL-II - A GPS data logger for birds. Memoirs of National Inst of Polar Research Special Issue.

[30] Afanasyev V, Prince PA (1993). A miniature storing activity recorder for seabird species. Ornis Scand.

[31] Phillips RA, Xavier JC, Croxall JP (2003). Effects of satellite transmitters on albatrosses and petrels. Auk.

[32] Dee DP, Uppala SM, Simmons AJ, Berrisford P, Poli P, Kobayashi S, Andrae U, Balmaseda MA, Balsamo G, Bauer P (2011). The ERA-interim reanalysis: configuration and performance of the data assimilation system. Q J R Meteorol Soc.

[33] Hooten MB, Johnson DS, McClintock BT, Morales JM. Animal movement: statistical models for telemetry data. New York: CRC Press; 2017.

[34] Pinheiro J, Bates D, DebRoy S, Sarkar D, Core R (2016). Team: nlme: linear and nonlinear mixed effects models. R package version.

[35] Pinheiro JC, Bates DM (2000). Mixed-effects models in S and S-PLUS.

[36] Nakagawa S, Schielzeth H (2013). A general and simple method for obtaining R2 from generalized linear mixed-effects models. Methods Ecol Evol.

[37] Yonehara Y, Goto Y, Yoda K, Watanuki Y, Young LC, Weimerskirch H, Bost C-A, Sato K (2016). Flight paths of seabirds soaring over the ocean surface enable measurement of fine-scale wind speed and direction. Proc Natl Acad Sci.

[38] Shaffer SA, Weimerskirch H, Costa DP (2001). Functional significance of sexual dimorphism in wandering albatrosses, *Diomedea exulans*. Funct Ecol.

[39] Richardson PL. Upwind dynamic soaring of albatrosses and UAVs. Prog Oceanogr. 2014;130:146-56.

[40] Nevitt GA, Losekoot M, Weimerskirch H (2008). Evidence for olfactory search in wandering albatross, Diomedea Exulans. Proc Natl Acad Sci U S A.

[41] Murray MD, Nicholls DG, Butcher E, Moors PJ, Walker K, Elliott G (2003). How wandering albatrosses use weather systems to fly long distances. 3. The contributions of Antarctic lows to eastward, southward and northward flight. Emu.

[42] Yu L, Jin X: Buoy perspective of a high-resolution global ocean vector wind analysis constructed from passive radiometers and active scatterometers (1987–present)**.** J Geophys Res. 2012;117,C11013. 10.1029/2012JC008069.

[43] Weimerskirch H, Bonadonna F, Bailleul F, Mabille G, Dell'Omo G, Lipp HP (2002). GPS tracking of foraging albatrosses. Science.

